# Pharmacokinetics, Pharmacodynamics, and Bioequivalence of Test Insulin Glargine Versus Reference Preparation (Lantus^®^) in Healthy Male Volunteers—By Euglycemic Clamp Technique

**DOI:** 10.3390/pharmaceutics17040418

**Published:** 2025-03-25

**Authors:** Zhongping Li, Min Liu, Yi Tao, Lei Wan, Yuan Chen, Mingxue Zhu, Hongtao Zhao, Chengyong Tang

**Affiliations:** 1 Phase I Clinical Trial Center, Bishan Hospital of Chongqing Medical University, Chongqing 402760, China; 13908392101@163.com (Z.L.); 17385120941@163.com (M.L.); roxray@163.com (L.W.); 15310579499@163.com (Y.C.); zhumingxueyee@163.com (M.Z.); zht18580747874@163.com (H.Z.); 2 Office of Academic Research, The First Affiliated Hospital of Chongqing Medical University, Chongqing 400016, China; taoziyi77@hotmail.com

**Keywords:** insulin glargine, pharmacokinetics, pharmacodynamics, euglycemic clamp, diabetes

## Abstract

The aim of this study was to evaluate the pharmacokinetics (PK), pharmacodynamics (PD), and safety of two insulin glargine preparations in healthy Chinese male subjects. **Methods:** Forty healthy Chinese male subjects were enrolled in this randomized, open, two-sequence, four-period, single-dose, crossover study and were randomly divided into RTRT or TRTR (first-period injection of test preparation, second-period injection of reference preparation, third-period injection of test preparation, fourth-period injection of reference preparation) groups. A 24 h euglycemic clamp test measured GIR. Plasma insulin glargine concentration and C-peptide were collected during the trial and analyzed by high-performance liquid chromatography–tandem mass spectrometry (HPLC-MS/MS) and enzyme-linked immunosorbent assay (ELISA). WinNonLin calculated PD/PK parameters and the equivalence of the two preparations was testified by SAS9.2. **Results:** The average concentration of C-peptide was lower than the baseline and the blood glucose was close to the targeted value in each sequence. PK parameters c_max_ of the test and the reference preparation insulin glargine were 0.580 and 0.614 ng·mL^−1^, and the AUC_0–24h_ were 9.782 and 10.436 h·ng·mL^−1^, respectively. PD parameters GIR_max_ were 42.748 and 45.279 mg·kg^−1^·min^−1^, and AUC_GIR,0–24h_ were 2.924 and 3.096 h·mg·kg^−1^·min^−1^, respectively. There was no clinically significant adverse reaction observed during the experiment. **Conclusions:** The glucose clamp has been established and bioequivalence between test preparation and reference preparation has been demonstrated.

## 1. Introduction

Diabetes is a global public health problem, and the prevalence of diabetes has increased significantly worldwide in the last 20 years or so. According to the International Diabetes Federation (IDF), there were approximately 537 million people with diabetes worldwide as of 2019, and this number is expected to reach 643 million by 2030 and 783 million by 2045 [1]. Insulin plays a huge role in controlling blood glucose and has been widely used in clinical practice. Insulin analogs are systematically categorized into five pharmacodynamic classes based on their pharmacokinetic (PK) profiles, and every class has distinct therapeutic roles, namely, prandial control (rapid/short-acting analogs target postprandial glucose excursions) and basal supplementation (long-acting analogs mimic physiological background insulin secretion) [2].

Insulin glargine, a long-acting insulin analog produced using recombinant DNA technology, is completely soluble in acidic solutions and has low solubility in neutral solutions [3]. After subcutaneous injection, the acidic solution in insulin is neutralized so that the insulin analog forms a precipitated microscopic deposit at the injection site, thus allowing for a slow and continuous release of insulin glargine from the injection site into the bloodstream, resulting in a smooth and predictable blood concentration without peaks for up to 24 h. Therefore, insulin glargine can be administered once a day and achieve good glycemic control [4,5,6]. Nowadays, as the market becomes increasingly saturated with emerging pharmaceutical products, rigorous evaluation of their clinical efficacy and safety profiles is critical.

The pharmacokinetic/pharmacodynamic (PK/PD) profile of insulin fundamentally determines its therapeutic application, administration regimen, and dosing optimization, directly impacting clinical efficacy and minimizing adverse events such as hypoglycemia [7]. Therefore, a thorough understanding of the pharmacokinetic/pharmacodynamic characteristics of insulin is beneficial for its application. The euglycemic clamp technique is recognized as the gold standard for evaluating insulin sensitivity of peripheral tissues [8]. The euglycemic clamp without hyperinsulinemia is used to keep blood glucose at slightly lower than normoglycemic levels by adjusting the intravenous glucose infusion rate (GIR) when the GIR can reflect the hypoglycemic effect of insulin preparations, i.e., pharmacodynamic characteristics [9]. In this study, we aimed to compare the pharmacokinetic characteristics of single-dose subcutaneous injection of the test preparation and the reference preparation of glargine insulin injection in Chinese healthy male subjects and evaluate the pharmacodynamic characteristics by glucose clamp technique [10] and, then, assess the bioequivalence and safety between the two preparations in Chinese healthy male subjects.

## 2. Materials and Methods

### 2.1. Study Drugs

Test preparation (T): insulin glargine injection (Lot. No. G01201712001), produced by Yichang HEC Changjiang Pharmaceutical Co., Ltd. (Yichang, China), specification 100 IU·mL^−1^, each 300 IU; reference preparation (R): insulin Glargine Injection (Lot. No. 8BJA025), produced by Sanofi Anwante (Beijing, China) Pharmaceutical Co., Ltd., specification for each 300 IU (pre-filled).

### 2.2. Study Subjects

Inclusion criteria: 18- to 45-year-old healthy male volunteers; body mass index (BMI) 19.0~24.0 kg m^−2^, and weight ≥ 50.0 kg; no smoking or alcohol addiction, no history of cardiovascular, liver, kidney, gastrointestinal, nervous, or endocrine system disease; normal glucose tolerance. Exclusion criteria: laboratory abnormalities or detection of the following diseases of clinical significance (including but not limited to gastrointestinal, renal, hepatic, neurological, hematological, endocrine, tumor, pulmonary, immune, psychiatric, or cardiovascular diseases); history of severe drug allergy, food allergy and drug dependence; use any prescription drugs, Chinese herbal medicine, over-the-counter drugs, health care products within 2 weeks before screening; participation in clinical trials or blood donation in the past 3 months; history of acupuncture and blood sickness, inability to tolerate venipuncture blood collection.

This trial was registered on 26 July 2019 with registration No. CTR20191031, which can be found at http://www.chinadrugtrials.org.cn (accessed on 30 October 2019). The study protocol was reviewed and approved by the Ethics Committee of the first affiliated hospital of Chongqing Medical University (NO. 20190101). All subjects signed written informed consent before participating in the study. Trial procedures were carried out in accordance with the Declaration of Helsinki and the principles of Good Clinical Practice.

### 2.3. Study Methods

#### 2.3.1. Total Design

This study was a randomized, open, two-preparation, four-period, two-sequence, single-dose, crossover trial, which was administered under fasting conditions and consisted of four periods with a washout period of 7 to 14 days between adjacent periods, i.e., the interval of administration between two successive periods was 7 to 14 days. Studies indicate that the intra-subject coefficient of variation (CV) for pharmacodynamic (PD) parameters of insulin glargine is higher than for pharmacokinetic (PK) parameters, with the CV for AUC_GIR,0–24h_ exceeding that of GIR_max_ and remaining ≤ 40% as reported in the literature [11,12,13]. Sample size estimation was based on the following parameters: T/R ratio: 95–105%; intra-subject CV: ~37%; significance level: 5% (two-sided); power: 80%; equivalence interval: 80–125%. Because this was a replicated four-period crossover design, the minimum sample size required was 32 subjects. To account for a 20% dropout/exclusion rate, 8 additional subjects were included, resulting in a final planned enrollment of 40 subjects. Based on the trial design, 40 eligible subjects were divided into the TRTR group (first-period injection of test preparation, second-period injection of reference preparation, third-period injection of test preparation, fourth-period injection of reference preparation) and RTRT group (first-period injection of reference preparation, second-period injection of test preparation, third-period injection of reference preparation, fourth-period injection of test preparation), 20 in each group.

#### 2.3.2. Clamping Test Process

The subjects ate at least 200 g of carbohydrates daily for 3 days before the clamp test to maintain weight stability. They were admitted to the Phase I clinical trial ward one day before each period of the test and were provided with a standard dinner and fasted overnight. The subjects fasted on the morning of the clamp test day, and the body weight was measured after urine evacuation; in the supine or sitting position, an indwelling catheter was placed on one side in the reverse cardiac direction to facilitate blood sampling, and a venous catheter was placed on the other side for infusion of 20% glucose solution. Venous blood was collected at 30, 20, and 10 min before subcutaneous injection of the reference preparation or the test preparation to determine the blood glucose level by automatic blood glucose and lactic acid analyzer (Germany, EKF Industrie, Elektronik GmbH, Barleben), which used a glucose oxidase method as the analysis method. The baseline blood glucose value was calculated as the average of the three blood glucose values, and the baseline blood glucose average minus 0.28 mmol·L^−1^ was used as the target blood glucose level of the subject in this clamp test. After subcutaneous injection of 0.4 IU·kg^−1^ test preparation or reference preparation in the morning of the test day of each period, PD blood samples were collected to detect the blood glucose level every 10 min from 0 to 8 h, every 20 min from 8 to 15 h, and every 30 min from 15 to 24 h with 4 mL each time. The infusion rate of 20% glucose solution was adjusted based on the blood glucose test results to maintain the blood glucose level of the subjects within the range of ±10% of the target blood glucose level, and the glucose infusion rate (GIR) was calculated, with the whole process lasting for 24 h.

#### 2.3.3. Detection of Insulin Glargine and C-Peptide

Blood samples of 4 mL and 2 mL were drawn at 20 min and 0 min before administration and 0.5, 1, 2, 3, 4, 5, 6, 8, 10, 12, 15, 18, 21, and 24 h after administration for quantification of plasma insulin glargine and serum C-peptide, respectively. The blood samples were centrifuged strictly according to the plan, and the plasma and serum were separated and stored in a refrigerator at −90 °C to −60 °C for the determination of plasma insulin glargine and serum C-peptide.

#### 2.3.4. Bioanalytical Methods

The concentrations of insulin glargine and its metabolites (M1 and M2) were detected by a validated high-performance liquid chromatography–mass spectrometry (HPLC–MS) method. The parameters were set as follows:

Chromatographic column: Waters, ACQUITY UPLC C18 (2.1 mm × 50 mm, 1.7 μm). Mobile phase: 0.5% acetic acid aqueous solution (A) and acetonitrile solution containing 0.5% acetic acid (B) for gradient elution. The initial flow rate was 0.60 mL·min^−1^ and the column temperature was 50 °C; the injection volume was 50 μL; the injector temperature was 5 °C.

Mass spectrometry conditions: electrospray ion source (ESI) [sprayer voltage 5500 V; temperature 550 °C; collider (N2) pressure 82 737.1 Pa; sprayer (N2) 413 685.4 Pa; curtain gas (N2) pressure 206 842.7 Pa; inlet potential (EP) 10 V]. Detection was performed in positive ion mode [the ion reactions for quantitative analysis were m/z 867.2→m/z 984.3 (insulin glargine), m/z 959.5→m/z 1118.7 (insulin glargine M1), m/z 959.5→m/z 1131.4 (insulin glargine M1), m/z 942.6→m/z 1 098.2 (insulin glargine M2), m/z 963.8→m/z 1123.5 (bovine insulin, internal standard)]. Plasma insulin glargine concentration was measured as the sum of the concentrations of the insulin glargine prototype drug, insulin glargine metabolite M1, and insulin glargine metabolite M2.

Serum C-peptide levels were determined by a validated enzyme-linked immunosorbent assay (ELISA) method. The ELISA kit was provided by American Laboratory Products Company, with the detection range of 40.0~2400 pmol·L^−1^.

#### 2.3.5. Safety Assessment

During the whole experiment and follow-up period, the safety was evaluated by vital signs, physical examination, 12-lead electrocardiogram, blood routine, urine routine, blood biochemistry, local injection reactions, and adverse events.

#### 2.3.6. Statistical Analysis

The performance quality of the clamp study was assessed in two ways. On the one hand, the level of glycemic control during the whole clamp was evaluated by calculating the mean difference, mean square deviation, and coefficient of variation of blood glucose concentration (CVBG) between the two preparations and the target blood glucose. On the other hand, the inhibition of serum C-peptide from both preparations was evaluated.

WinNonLin version 7.0 (pharsight Corporation, Sunnyvale, CA, USA) was used to calculate the pharmacodynamic and pharmacokinetic parameters by a non-compartmental model. The primary pharmacokinetic (PK) parameters included maximum plasma concentration (c_max_), the area under the plasma concentration versus time curve (AUC_0–24h_), and time to reach c_max_ (t_max_). The primary pharmacodynamic (PD) parameters were the area under the glucose infusion rate versus time curve (AUC_GIR,0–24h_), the peak of glucose infusion rate (GIR_max_), and time to GIRmax (tGIR_max_). Statistical analyses of data were performed in SAS^®^ version 9.4 (SAS Institute Inc., Cary, NC, USA). PK and PD parameters were summarized with descriptive statistics and presented as arithmetic mean (standard deviation, SD) or geometric mean (coefficient of variation, CV), except for *t*_max_ and tGIR_max_, which were performed as median (range). Log-transformed PK and PD parameters were assessed by a linear mixed effects model with treatment, period, and sequence as fixed effects, while the subject within sequence and treatment was treated as a random effect. Based on the average bioequivalence method, the main PK parameters or PD parameters were transformed by natural logarithm, and the bioequivalence was compared by calculating the 90% confidence interval (90% CIs) of the geometric mean ratio (T/R) of each parameter. In addition, the *t*_max_ and *t*GIRmax of the two formulations were compared by the nonparametric Wilcoxon rank-sum test. The general steps for bioequivalence calculation are as follows:

1. Log-Transform PK Parameter: log-transforming AUC and Cmax
2. Check Normality Assumptions: Validate the normality of log-transformed parameters using the Shapiro–Wilk test.
3. Fit a Mixed Model: Use a linear mixed effects model to account for crossover effects (sequence, period, treatment, and subject variability).
4. Calculate the Geometric Mean Ratio (GMR): Extract the mean difference (estimate) for test vs. reference, computing the 90% CI on the log scale and exponentiate the values to convert to the original ratio scale after running the model.

## 3. Results

### 3.1. Basic Information of Subjects

A total of 40 subjects were enrolled in this study. One subject withdrew from the trial due to mild adverse events before the first administration, and 39 subjects completed at least one period of administration. One of them withdrew after only injected reference preparation and two of them withdrew after completing the first three periods both came from the RTRT sequence. The demographic details of the TRTR and RTRT groups are presented in Table 1. There was no significant difference in demographic data between the two sequences (*p* > 0.05).

### 3.2. Quality Evaluation of Clamp Test

#### 3.2.1. Blood Glucose Level

During the test, the blood glucose levels of most of the test points among all subjects were maintained within the range of ±10% of the target blood glucose value, as shown in Table 2. The difference between the post-dose blood glucose (BG) and the target BG was slight; CVBG was relatively low and comparable in all groups, which indicated that the fluctuation of blood glucose was very small, and the clamp platform was stable.

#### 3.2.2. C-Peptide Inhibition

**Figure 1:**
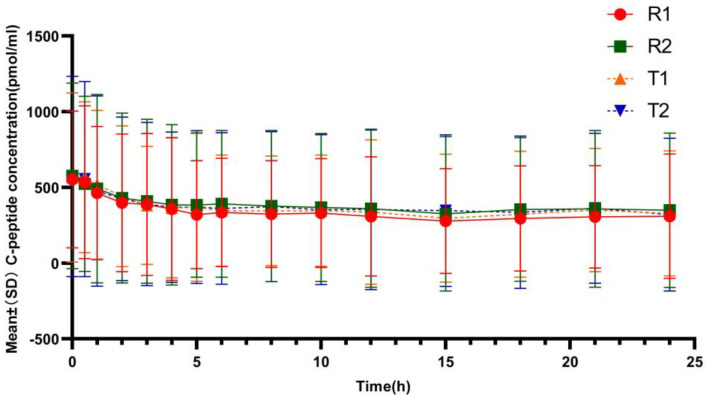
Mean C-peptide concentration reduction–time profiles after subcutaneous injection of the test and reference insulin glargine. (T1/R1 and T2/R2 represent the groups receiving the test/reference preparation for the first and second time, respectively).

Figure 1 showed that the mean serum C-peptide concentration at each analysis time point in each period was lower in the subjects after receiving the test or reference preparations than before administration, suggesting that the secretion of endogenous insulin was suppressed in all subjects during clamp. The mean ratios of C-peptide reduction were 35.30%/36.10% in the test group (T1/T2) and 38.55%/35.12% in the reference group (R1/R2), indicating comparable degree of suppression of the endogenous insulin between the two formulations.

### 3.3. Pharmacodynamic Evaluation

**Figure 2:**
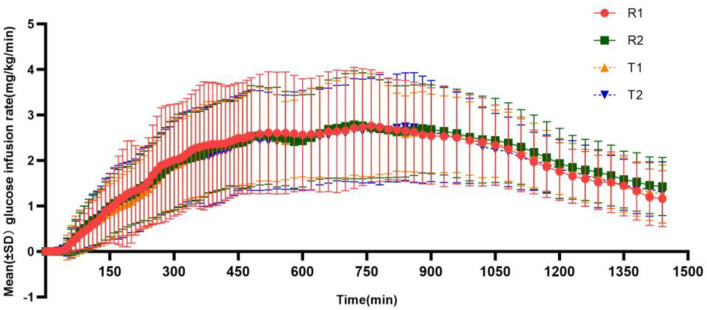
Mean glucose infusion rate (GIR)–time curves for the test and reference insulin glargine. (T1/R1 and T2/R2 represent the groups receiving the test/reference preparation for the first and second time, respectively).

After subcutaneous injection of insulin glargine test or reference preparation, as shown in Figure 2, the GIR values gradually increased, but the effect was gentle and with no obvious peak infusion rate, and the average glucose infusion rate of the test preparation or the reference preparation was relatively consistent. The main pharmacodynamic parameters of the test and reference preparations are summarized in Table 3.

### 3.4. Pharmacokinetic Assessment

**Figure 3:**
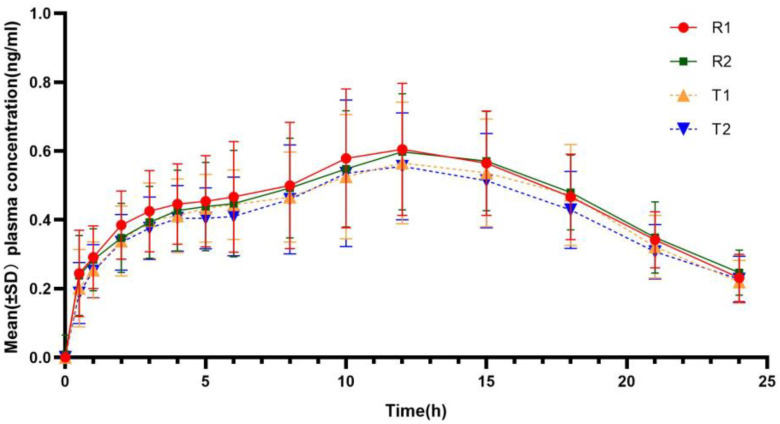
Mean insulin concentration–time curves for the test and reference insulin glargine. (T1/R1 and T2/R2 represent the groups receiving the test/reference preparation for the first and second time, respectively).

The mean insulin glargine profiles after subcutaneous injection of 0.4 IU·kg^−1^ were similar between the two formulations (Figure 3), with a median time to reach maximum concentration at 12 h, which was close to the median time to peak of glucose infusion rate (tGIRmax). The main pharmacokinetic parameters of plasma insulin glargine are shown in Table 3.

### 3.5. Bioequivalence Evaluation

The results of the bioequivalence analysis of test and reference preparations are shown in Table 4. The 90% CIs of the GMRs (T/R) for the main pharmacokinetic parameters (Cmax and AUC0–24 h) and pharmacodynamic parameters (AUCGIR,0–24 h, and GIRmax) after natural logarithm conversion all fell within the range of 80% to 125%, which demonstrated bioequivalence of two preparation in healthy Chinese subjects. In addition, the difference in Tmax and tGIRmax between the two insulin formulations was not statistically significant (*p* > 0.05). The intra-subject variability in the PK and PD parameters was also comparable between the two formulations.

### 3.6. Safety Evaluation

A total of 42 drug-related adverse events occurred in 14 subjects, of which 18 occurred in 9 (24%) subjects with the test formulation and 24 in 11 (28%) subjects with the reference formulation (Supplementary Table S1). The severity of these adverse reactions was mostly mild, except for one moderate case. The common (incidence ≥ 10%) adverse events associated with the test drug were hypoglycemia and elevated blood bilirubin, and they were temporary. A total of 24 cases of hypoglycemia occurred in 6 subjects (15.4%). Elevated serum bilirubin occurred in 4 subjects (10.3%) with 7 cases. No clinically significant hypoglycemia, severe hypoglycemia, severe adverse event, serious adverse event, allergic reactions, or adverse reactions at the injection site were observed in this study. The safety of the test preparation and the reference preparation were similar and both were well tolerated.

## 4. Discussion

This study was designed as a two-preparation, four-period, two-sequence, single-dose, crossover trial. Through the cross-control of the subjects themselves, the sample size was reduced while minimizing intra-individual variability [14]. Considering that large inter-individual variability may affect the metabolic effects of the study drug, healthy male volunteers were selected for this study rather than T1DM patients [15]. According to previous glargine clamp studies and EMEA guidelines and considering the information of reference preparation instructions and the requirements of regulations, the safety of clinical medication, and the feasibility of sample test, the dose of insulin glargine in this study was 0.4 IU·kg^−1^ [9,11,12,13,15]. A washout period of 7–14 days was set to avoid any residual effects. With the consideration of tolerance of subjects and the known duration of action of insulin glargine, the duration of the clamp was set at 24 h to avoid prolonged fasting of the healthy volunteers and to mimic the dosing interval, which was similar to the previous studies in healthy volunteers [16,17]. It was also consistent with the recommended clamp duration (at least 24 h) of the EMA guidelines 15 for intermediate- and long-acting insulin.

In this study, we used the non-hyperinsulinemic-euglycemic clamp technique for evaluating the pharmacodynamic characteristics. One study compared the effects of the euglycemic clamp test with and without a hyperinsulinemic platform on the PK and PD parameters of insulin preparations. It was found that the hyperinsulinemic-euglycemic clamp test would cause excessive insulin levels in the circulation and exaggerate the PK data of injected insulin; the existing hyperinsulinemia might partially mask and delay the actual peak time of the tested insulin preparation. During the clamping process, the blood glucose was maintained below the fasting blood glucose level, which could not only inhibit the secretion of endogenous insulin but also prevent the secretion of glucose-increasing hormones. Therefore, the PK/PD evaluation of insulin preparations performed with a euglycemic clamp was preferable to with a hyperinsulinemic-euglycemic clamp in healthy male subjects [15]. At present, the quality of clamp study performance is mainly evaluated by blood glucose level and plasma C-peptide level [18]. EMA recommends that the mean, root-mean-square deviation, and coefficient of variation of blood glucose concentrations (CVBG) should be calculated to assess the performance of clamp studies [15]. Previous studies [19,20] considered that CVBG less than 4.5% or 5% indicated a good quality. During our whole experiment, the blood glucose level of the subjects was always controlled within the target range with CVBG below this standard. And the C-peptide was also maintained below the baseline level, which reflected the inhibition of endogenous insulin secretion. Thus, the quality of the clamp test was good, and the results of pharmacodynamic evaluation parameters were reliable. Additionally, some studies [21,22] have suggested that C-peptide inhibition of more than 50% can more strongly indicate freedom from endogenous insulin interference. Although the mean ratios of C-peptide reduction in this study were 35.30%/36.10% in the test group (T1/T2) and 38.55%/35.12% in the reference group (R1/R2), the C-peptide concentrations of the two preparations were comparable at all times, and the bioequivalence of PK/PD could still be well evaluated. In this study, blood drug concentration was detected by high-performance liquid chromatography–tandem mass spectrometry, which is a method of combining high-efficiency separation and multi-component characterization and quantification with the characteristics of high sensitivity and strong selectivity, a simpler method with better accuracy [23]. After subcutaneous injection, insulin glargine is rapidly metabolized into active metabolites M1 and M2. M1 is the main metabolite, accounting for more than 90% of the circulating active components of insulin glargine. Therefore, in this trial, the PK parameter characterized the sum of the concentrations of M1, M2, and the insulin glargine prototype drug.

According to the relevant guidelines of bioequivalence statistics [24], combined with the results of intra-subject variability (within 30%) of the main PK and PD parameters of the reference preparation in this study, the average bioequivalence methodology was used to evaluate the bioequivalence of reference and test preparations. The absorption rate and absorption degree of the reference and the test preparations in the human body were similar, and the main PK parameters were comparable. In addition, the PK parameters cmax and AUC_0–24h_ obtained in this trial in Chinese healthy adult male subjects injected with insulin glargine were similar to those reported in similar studies at home and abroad [16] and the reference formulation instructions [25]. The tmax (median) of the two preparations was 12 h, which was consistent with the literature [16,17,26,27] and the instructions of the reference preparation. The median of tGIR_max_ of the subjects injected with insulin glargine test preparation in each period was close to the data reported in the literature [12,26]. In addition, this study showed that the PK parameters and PD parameters of the test and reference preparations had similar and relatively low intra-subject variability, which may be due to the reliable clamping platform and the strict selection of subjects. Finally, the hypoglycemic effects of the two preparations were similar (AUC_GIR, 0–24h_, GIR_max,_ and tGIR_max_ were all comparable), and the 90% CIs of the geometric mean ratio for the main PK and PD parameters of the test to reference preparations by natural logarithm transformation fell within the range of 80% to 125% [24] making both preparations bioequivalent.

## 5. Conclusions

In summary, this trial evaluated the pharmacodynamic and pharmacokinetic characteristics of the two glargine insulin injections in healthy Chinese male subjects and the bioequivalence between the two formulations. After subcutaneous injection, the serum insulin concentration and glucose infusion rate (GIR) were monitored to evaluate the PK/PD parameters. The 90% CIs of the GMRs for the main parameters of PK and PD of the reference and test preparations were in the acceptable range (80~125%). In terms of safety, the incidence rates of adverse events for the two formulations were similar (24% vs. 28%). No serious adverse reactions were reported. Those results suggest that the two preparations were bioequivalent and had a good safety profile.

## Figures and Tables

**Table 1:** Demographic characteristics of subjects.

Characteristics	TRTR (N = 19)	RTRT (N = 20)	Total (N = 39)
Age (year)	26.5 (±4.48)	28.7 (±5.74)	27.6 (±5.21)
Hight (cm)	169.21 (±6.04)	169.88 (±5.41)	169.55 (±5.66)
Weight(kg)	62.95 (±5.30)	64.30 (±5.73)	63.64 (±5.49)
BMI (kg/m^2^)	21.98 (±1.24)	22.25 (±1.20)	22.12 (±1.21)

**Table 2:** Indicators related to blood glucose during the clamp test.

Indicators	Arithmetic Mean (SD)			
	T1 (*n* = 38)	T2 (*n* = 36)	R1 (*n* = 39)	R2 (*n* = 38)
Mean pre-dose BG/mmol·L^−1^	5.31 (0.34)	5.32 (0.33)	5.29 (0.34)	5.36 (0.30)
Target BG/mmol·L^−1^	5.03 (0.34)	5.04 (0.33)	5.01 (0.34)	5.08 (0.30)
Mean post-dose BG/mmol·L^−1^	5.04 (0.32)	5.05 (0.31)	5.03 (0.33)	5.07 (0.28)
Difference between the mean postdose BG and the target BG/mmol·L^−1^	0.01(0.04)	0.02 (0.06)	0.01(0.06)	0.00 (0.03)
CVBG (%)	3.58	3.55	3.73	3.58

BG, blood glucose; CVBG, coefficient of variation of blood glucose concentration. CVBG was calculated based on the post-dose BG. T1/R1 and T2/R2 represent the groups receiving the test/reference preparation for the first and second time, respectively.

**Table 3:** Pharmacokinetic parameters and pharmacodynamic parameters.

	T Geo Mean (Geo CV, %)		R Geo Mean (Geo CV, %)	
Parameters (Unit)	T1 (*n* = 38)	T2 (*n* = 36)	R1 (*n* = 39)	R2 (*n* = 38)
PD parameters				
GIR_max_ (mg/kg/min)	2.912 (38.22)	2.890 (39.14)	3.067 (42.69)	3.116 (34.50)
*t*GIR_max_ (h) ^a^	11.17 (5.50, 24.00)	12.00 (4.83, 22.00)	10.67 (3.17, 19.00)	12.33 (3.67, 18.50)
AUC_GIR,0–24h_ (h·mg/kg/min)	42.368 (40.85)	42.577 (42.62)	43.849 (46.21)	46.635 (34.94)
AUC_GIR,0–12h_ (h·mg/kg/min)	18.086 (58.51)	18.646 (50.96)	20.491 (53.56)	20.383 (44.37)
AUC_GIR,12–24h_ (h·mg/kg/min)	23.658 (36.39)	23.631 (40.02)	24.005 (36.06)	25.746 (34.42)
PK parameters				
*C*_max_ (ng/mL)	0.587 (28.79)	0.570 (31.57)	0.618 (31.59)	0.609 (28.85)
AUC_0–24h_ (h·ng/mL)	9.919 (27.06)	9.587 (25.41)	10.494 (26.37)	10.364 (27.20)
AUC_0–12h_ (h·ng/mL)	4.899 (27.51)	4.766 (29.28)	5.229 (32.30)	5.042 (31.30)
AUC_12–24h_ (h·ng/mL)	4.980 (29.81)	4.791 (23.75)	5.209 (23.84)	5.275 (26.37)
T_max_ (h) ^a^	12.00 (4.00, 18.01)	12.00 (4.01, 15.03)	12.00 (4.00, 18.00)	12.00 (3.00, 18.01)
*t*_1/2_ (h) ^b^	5.81 (±2.78)	7.60 (±4.76)	6.62(±4.29)	7.05 (±4.26)

Geo Mean: geometric mean; geo CV: geometric coefficient of variation. ^a^ T_max_ and ^a^ tGIR_max_ were presented as median (minimum, maximum). ^b^ *t*_1/2_ was presented as the arithmetic mean (±standard deviation). T1/R1 and T2/R2 represent the groups receiving the test/reference preparation for the first and second time, respectively.

**Table 4:** A comparison of the main pharmacokinetic and pharmacodynamic parameters between the test and reference preparations.

Parameters	GM				Intra-Subject Variability (%)	
	T (*n* = 36)	R (*n* = 38)	GMR (%)	90%CI of GMR	T	R
PD parameters						
AUC_GIR, 0–24 h_ (h·mg/kg/min)	42.748	45.279	94.41	(88.33, 100.91)	25.35	25.32
GIR_max_ (mg/kg/min)	2.924	3.096	94.45	(88.78, 100.48)	23.42	23.18
PK parameters						
AUC_0–24 h_ (h·ng/mL)	9.782	10.436	93.74	(88.84, 98.91)	18.90	22.16
*c*_max_ (ng/mL)	0.580	0.614	94.56	(88.86, 100.63)	21.39	25.30

GM, geometric mean; GMR, geometric mean ratio.

## Data Availability

The data presented in this study are available on request from the corresponding author due to (specify the reason for the restriction).

## Supplementary Materials

The following supporting information can be downloaded at: https://www.mdpi.com/article/10.3390/pharmaceutics17040418/s1.
