# Supplementary material for: Pharmacokinetics, Pharmacodynamics, and Bioequivalence of Test Insulin Glargine Versus Reference Preparation (Lantus®) in Healthy Male Volunteers—By Euglycemic Clamp Technique
Source: Pharmaceutics. 2025 Mar 25;17(4):418. doi: 10.3390/pharmaceutics17040418 (PMC12030383; doi:10.3390/pharmaceutics17040418)
Supplement: Supplementary file 1 [file pharmaceutics-17-00418-s001.zip › pharmaceutics-3492792-supplementary.pdf]

Supplementary Table 1. Adverse Events of insulin glargineTest and Reference

System Organ Classification/Preferred Term	T (N = 38) N (%)/m	R (N = 39) N (%)/m	Total (N = 39) N (%)/m
**Examinations**	5(13.2)/6	5(12.8)/7	6(15.4)/13
Increased blood lactate dehydrogenase	0/0	1(2.6)/1	1(2.6)/1
Increased serum creatinine	1(2.6)/2	1(2.6)/1	1(2.6)/3
Elevated blood bilirubin	3(7.9)/3	3(7.7)/4	4(10.3)/7
Abnormal T wave	1(2.6)/1	1(2.6)/1	1(2.6)/2
**Metabolic and nutritional diseases**	5(13.2)/11	3(7.7)/13	6(15.4)/24
Hypoglycemia	5(13.2)/11	3(7.7)/13	6(15.4)/24
**Cardiac organ diseases**	0/0	3(7.7)/4	3(7.7)/4
Sinus bradycardia	0/0	3(7.7)/4	3(7.7)/4
**Diseases of blood vessels and lymphatics**	1(2.6)/1	0/0	1(2.6)/1
Hypotension	1(2.6)/1	0/0	1(2.6)/1
**Total**	9(23.7)/18	11(28.2)/24	14(35.9)/42
